# Plasma concentration of brominated flame retardants and postmenopausal breast cancer risk: a nested case-control study in the French E3N cohort

**DOI:** 10.1186/s12940-020-00607-9

**Published:** 2020-05-20

**Authors:** Francesca Romana Mancini, German Cano-Sancho, Oceane Mohamed, Iris Cervenka, Hanane Omichessan, Philippe Marchand, Marie-Christine Boutron-Ruault, Patrick Arveux, Gianluca Severi, Jean-Philippe Antignac, Marina Kvaskoff

**Affiliations:** 1 CESP, Faculté de médecine, Université Paris-Saclay, UVSQ, INSERM, Villejuif, France; 2 Gustave Roussy, Villejuif, France; 3 LABERCA, Oniris, INRAE, Nantes, France; 4 Breast and Gynaecologic Cancer Registry of Côte d’Or, Georges-François Leclerc Cancer Centre, UNICANCER, Dijon, France; 5 Departement of Statistics, Computer Science and Applications (DISIA), University of Florence, Florence, Italy

**Keywords:** Breast cancer, Brominated flame retardants (BFRs), Pentabromodiphenyl ethers (PBDEs), Polybrominated biphenyls (PBBs), E3N cohort, Biomonitoring

## Abstract

**Background:**

Brominated flame retardants (BFRs) are lipophilic substances with endocrine-disrupting properties. To date, only few investigations, mainly retrospective case-control studies, have explored the link between internal levels of BFRs and the risk of breast cancer, leading to conflicting results. We investigated the associations between plasma concentrations of two main groups of BFRs, PBDEs (pentabromodiphenyl ethers) and PBBs (polybrominated biphenyls), and the risk of breast cancer in a nested case-control study.

**Methods:**

A total of 197 incident breast cancer cases and 197 controls with a blood sample collected in 1994–1999 were included. Plasma levels of PBDE congeners (BDE-28, BDE-47, BDE-99, BDE-100, BDE153, BDE-154) and of PBB-153 were measured by gas chromatography coupled to high-resolution mass spectrometry. Conditional logistic regression models, adjusted for potential confounders, were used to estimate odds ratios (ORs) and 95% confidence intervals (CIs).

**Results:**

Women were aged 56 years on average at blood draw. All cases, except for one, were diagnosed after menopause, with an average age at diagnosis of 68 years. Overall, we found no evidence of an association between plasma levels of PBDEs and PBB-153 and postmenopausal breast cancer risk (log-concentrations of BFRs yielding non-statistically significant ORs of 0.87 to 1.07). The analysis showed a non-linear inverse association for BDE-100 and BDE-153 and postmenopausal breast cancer risk; nevertheless, these findings were statistically significant only when the exposure was modeled as ng/L plasma (third vs. first quintile: OR = 0.42, 95%CI = 0.19–0.93 and OR = 0.42, 95%CI = 0.18–0.98, respectively) and not when modeled as ng/gr of lipids (OR = 0.58, 95%CI = 0.27–1.25 and OR = 0.53, 95%CI = 0.25–1.17). These results were unchanged in stratified analyses by tumor hormone receptor expression or body mass index.

**Conclusions:**

Our results suggest no clear association between internal levels of PBDEs and PBB-153 and the risk of breast cancer in postmenopausal women. However, these findings need to be carefully interpreted, taking into account limitations due to the limited number of women included in the study, the lack of information concerning genetic susceptibility of cases, and the unavailability of exposure assessment during critical windows of susceptibility for breast cancer. More studies are warranted to further investigate the relationships between PBDE and PBB exposure and breast cancer risk.

## Introduction

Despite a large body of research, the etiology of breast cancer has not yet been fully delineated, as established risk factors cannot solely explain the increased incidence reported worldwide. In Europe, breast cancer is the most common cancer in women with an incidence of 60 to 155 cases per 100,000 [1]. The main known risk factors for breast cancer include non-modifiable risk factors - such as age, early menarche, late menopause, family history of breast cancer, and genetic mutations (e.g. mutations in the *BRCA1* and *BRCA2* genes) - and modifiable risk factors - such as use of hormonal treatments, alcohol consumption, tobacco smoking, overweight, and lack of physical activity, [2]. Nevertheless, it has been estimated that less than 25% of breast cancers are attributable to the main modifiable risk factors and between 10 and 30% to hereditary factors [3, 4]. There is growing concern that exposure to chemical environmental contaminants, particularly endocrine-disrupting chemicals (EDCs), could be one of the factors that led to an increased incidence of breast cancer in the Western world [5].

Brominated flame retardants (BFRs) are lipophilic substances present as a mixture of congeners in several consumer products to make them less flammable. Produced since the early 1960s, their use has been progressively limited and banned in Europe during the 90s when the production reached the levels of 33,000 metric tonnes in 1994 [6]. PBBs were first restricted by the 4th amendment to the marketing and use Directive 76/769/EEC adopted in 1984 and in 2000 the production was voluntarily ceased by the industry [7]. Commercial mixtures Penta- and Octa-PBDE products were first restricted in 1976 under the Directive 76/769/ECC and banned in 2003 in concentrations higher than 0.1% by mass [8]. Nevertheless, due to their resistance to degradation, BFRs are widespread in the environment, even in areas located remotely from sites of production or use [9]. Diet has been identified as the main source of exposure to BFRs for adults, although the exposure can also be related to other routes such as inhalation or dermal absorption as a consequence of their accumulation in indoor dust [10]. Unfortunately, the long-term toxic effects of these BFRs in humans are not completely known, although they are suspected to act as EDCs, since a link between exposure to BFRs and several endocrine-related diseases has been reported [11]. In 2019, PBDEs, one of the major groups of BFRs, have been included in the high-priority list of agents not previously evaluated by the International Agency for Research on Cancer (IARC) Monographs on the basis of relevant bioassay and mechanistic evidence [12].

There is increasing interest in understanding the contribution of BFRs to breast cancer incidence; nevertheless, evidence of an association between environmental exposure to these chemicals and breast cancer risk is still inadequate to draw firm conclusions [5, 13]. To date, only few studies have investigated the link between internal levels of BFRs and the risk of breast cancer, with inconsistent results. To the best of our knowledge, regarding PBDE, all available studies followed a retrospective case control study design [14–17], while for PBB the available studies were case-control studies nested in a prospective cohort [18–20]. Finally, all the currently available studies have been conducted in the US [14–16, 18–20] or in China [17], and none was performed in Europe.

Our objective was to prospectively investigate the associations between plasma concentrations of two main groups included in the family of BFRs, PBDEs and PBBs, and the risk of postmenopausal breast cancer in women in a case-control study nested in the French E3N cohort.

## Methods

### The E3N study

E3N (*Etude Epidémiologique auprès de femmes de l’Education Nationale*) is a prospective cohort study involving 98,995 French women, aged 40–65 years at inclusion in 1990 and insured by a national health insurance covering workers from the French National Education System (*Mutuelle Générale de l’Education Nationale*, MGEN) [21]. Participants were enrolled after completing a baseline self-administered questionnaire and returning a signed informed consent. Follow-up questionnaires were sent every 2–3 years thereafter. Detailed cancer risk factor data were collected through questionnaires at different time points during follow-up, including reproductive history, use of hormonal treatments, anthropometric characteristics, smoking habits, alcohol consumption, diet, and physical activity. The average follow-up rate per questionnaire cycle has been of 83%, and to date, the total loss to follow-up since 1990 has been < 3%. The study was approved by the French National Commission for Data Protection and Privacy.

Between 1994 and 1999, E3N participants were invited to donate blood, resulting in the collection of blood samples from approximately 25,000 participants. Each sample was separated into 28 aliquots (i.e. plasma, serum, buffy-coat, leukocytes, and erythrocytes) that were stored in plastic straws in liquid nitrogen containers (− 196 °C) in a biobank.

Breast cancer cases were identified through self-report in the questionnaires, from the MGEN files, or through information from death certificates. Deaths were reported by family members and by searches in the MGEN files, and causes of death were obtained from the National Death Index. Pathology reports were obtained for 93% of incident cases. We also considered cases for which pathology reports have not been obtained, because the proportion of false-positive self-reports was low in our study population (< 5%). Cases were identified up to 2013, which was therefore used as the date of end of follow-up in statistical analyses.

### The nested case-control study on breast cancer

As previously described [22], 281 breast cancer cases for which at least 3 aliquots of serum and 6 aliquots of plasma were available in the biobank were identified among the E3N population. From these, all cases who had not completed the dietary questionnaire in 1993 (*n* = 27) or who were diagnosed before blood sampling and/or before returning the dietary questionnaire were excluded (*n* = 11). Cases of Paget’s disease and benign breast disease were also excluded (*n* = 3). Finally, 240 incident breast cancer cases were available. Due to budget constraints, among those, 197 incident breast cancer cases (174 invasive and 23 in situ) were randomly selected and included in the study.

For each case, one control was sampled from women who were free of breast cancer at the time of diagnosis of the corresponding case (density sampling method). Controls were matched to cases by age (±2 years), menopausal status (premenopausal or postmenopausal), body mass index (BMI) (< 25 or ≥ 25 kg/m^2^), all at blood collection, and year of blood collection.

#### Measurement of biomarkers of exposure

The methodologies applied to isolate, detect, and quantify the PBDE congeners (BDE-28, BDE-47, BDE-99, BDE-100, BDE-153, BDE-154) and PBB-153 have been published elsewhere [23, 24]. Briefly, plasma samples were first submitted to a liquid/liquid extraction with pentane. Resulting extracts were weighed to measure fat content using an enzymatic method (Biolabo,Maizy, France), and reconstituted in hexane for further purification. The determinations were performed by gas chromatography (Agilent 7890A) coupled to high-resolution mass spectrometry (GC-HRMS) on double sector instruments (JEOL MS 700D and 800D) after electron impact ionization (70 eV), operating at 10,000 resolutions (10% valley) and in the single ion monitoring (SIM) acquisition mode. All the analyses have been conducted in an ISO 17025:2005 accredited laboratory. The limits of detection (LOD) were 0.03 ng/L for BDE-28, 0.02 ng/L for BDE-154, and 0.1 ng/L for BDE-47, BDE-99, BDE-100, BDE-153 and PBB-153. To ensure the quality of the analysis, besides the appropriate use of internal standards in each sample, ^13^C_12_ labelled standards were added at the end of each clean-up process in order to calculate recoveries that ranged between 60 and 120%. The accuracy was determined according the ISO 17025 requirements, setting up the lower spiking level at 0.5 ng/L (*n* = 5), being the trueness within the range between 3% (BDE-99) and 37% (BDE-28), and the precision ranged between 13% (BDE-47) and 28% (PBB153). The analytical performance was considered acceptable for all congeners; however, it should be noted the analytical uncertainty associated to lower concentrations for BDE28. Moreover, cleaned laboratory glassware was rinsed with dichloromethane prior to use and the analyses were carried out in an over-pressurized room to minimize environmental contamination followed by a continuous monitoring of the analytical procedure implemented through procedural blanks [25]. The blank controls were not deducted because of the absence of analytical contamination. The accuracy of the analytical method was further ensured by regular participation of the laboratory to proficiency tests organized by the European Reference Laboratory (EURL) for POPs. The total plasma lipid (TPL) levels were calculated by using the Akins formula, combining the concentration of phospholipids (PHO), triacylglycerides (TAG), total cholesterol (t.CHO) and free cholesterol (f.CHO) as follows: TPL = 1.677*(t.CHO-f.CHO) + f.CHO + TAG+PHO) [26].

#### Statistical analysis

In all statistical analyses, values below the limit of detection (LOD) were substituted with the equivalent of ½LOD. Kruskal–Wallis tests were performed to compare concentrations of PBDEs and PBB-153 between cases and controls. Spearman rank correlation tests were performed to determine correlation structures between plasma concentrations of PBDEs and PBB-153.

Conditional logistic regression models were used to estimate odds ratios (ORs) and 95% confidence intervals (CIs) of breast cancer in relation to plasma concentration levels of PBDEs and PBB-153 expressed as ng/L plasma. We also used conditional logistic regression models using both the exposure variable as a continuous variable, after log-transformation, and as a categorical variable, dividing it into quintile groups based on plasma concentrations of PBDEs and PBB-153 in controls. When considering the exposure as a categorical variable, tests for linear trend were performed by assigning the median value to each quintile group and by modeling these values as continuous variables. For all adjustment variables, we considered the value collected in the last questionnaire available before the date of diagnosis in cases. All analyses were performed for each substance separately and for the sum of concentrations of PBDEs. An unadjusted model (Model 0) was fitted, as well as three adjusted models with an increasing number of covariates. Model 1 was adjusted for total plasma lipid content by the addition of a separate term in the model (ng/L, continuous), as recommended by Schisterman et al. [27]. Model 1 was further adjusted for smoking status (never vs. ever), physical activity measured in metabolic equivalent tasks (MET)-hour/week (< 35 vs. ≥35), education (≤ 12 years, 12 to 14 years, > 14 years), personal history of benign breast disease (no vs. yes), and family history of breast cancer (none, in first-degree relatives, in extended relatives), parity and age at first full-term pregnancy (FFTP) (no children, 1 or 2 children and < 30 years old at FFTP, ≥3 children and < 30 years old at FFTP, ≥ 30 years old at FFTP), total breastfeeding duration (never, ≤6 or > 6 months), age at menarche (< 13 years vs. ≥ 13 years), current use of menopausal hormone therapy (pre-menopausal, yes, no), use of oral contraceptives (ever vs. never), menopausal status and age at menopause (pre-menopausal, menopause before age 51, menopause at age 51 or later). Finally, Model 2 was further adjusted for adherence scores to the Healthy and the Western dietary patterns (above vs. below the median), both derived using principal component analysis, as previously described [28]. The selection of confounders was done a priori, based on the known breast cancer risk factors available in our dataset that are potentially associated with exposure to PBDEs and PBB-153.

Using multinomial regression, stratified analyses were performed based on tumor expression of estrogen (ER– vs. ER+) and progesterone (PR– vs. PR+) receptors including all variables used in Model 2. Since multinomial regression models do not consider the case-control matching, multinomial models were additionally adjusted for the matching criteria: age at blood draw (continuous), BMI at blood draw (continuous), and year of blood draw (continuous), except for menopausal status at blood draw, in order to avoid over-adjustment considering the a priori inclusion of age at menopause in the model (premenopausal, postmenopausal and age at menopause < 51 years, postmenopausal and age at menopause ≥51 years). All subjects for whom the information on hormone receptors was not available were grouped as one “missing” category and inserted in the model.

In order to better understand the underlying association between lipids, BFRs and breast cancer, all analyses were repeated using concentrations of PBDEs and PBB expressed as ng/gr of lipids in the plasma.

Finally, the analyses were repeated excluding the case diagnosed before menopause and her matched control.

#### Sensitivity analysis

Other authors highlighted that the evolution of body size over lifetime may have an impact of the risk of developing breast cancer [29]. On the other hand, because of the high lipophilicity of BFRs, it has been hypothesized that changes in body weight prior to blood sampling may partly explain circulating levels of BFRs, and that their absorption, distribution, metabolism, and excretion can be influenced by the evolution of body size [30]. In order to test this, we built two additional models in a sensitivity analysis. First, Model 2 was additionally adjusted for the evolution of women’s body size between ages 8 and 35 years: the evolution trajectories of body size were determined from the 8 somatotypes of Sorensen reported by the women at recruitment [31]. The women reported which somatotypes best reflected their body size at different periods in life (“around 8 years old”, “at puberty”, “around 20-25 years old”, “around 30-35 years old”). Using the PROC TRAJ of SAS 9.3, we highlighted three trajectories of evolution of the body size of each woman using age as time scale: normal body size from ages 8 to 35 years; normal body size until puberty, followed by a large body size afterwards; and large body size from ages 8 to 35 years. All women were included in these trajectories.

Then, Model 2 was adjusted for BMI variation over the 5 years preceding blood sampling (BMI decrease of more than 2 points; stable BMI; and BMI increase of more than 2 points).

We conducted a stratified analysis based on BMI, considering groups with BMI < 25 and  ≥25 kg/m^2^.

In our study population, missing values were < 5% for all variables and were imputed to the median (continuous variables) or modal value (categorical variables).

Statistical analyses were performed using the SAS software (version 9.4, SAS Institute). The threshold for statistical significance was set at 5%. All statistical tests were two-sided.

## Results

The main characteristics of the study population are presented in Table 1.

**Table 1:** Distribution of selected characteristics for 394 study participants, E3N cohort

	All	Control	Case
	N(%) or Mean (STD)	N(%) or Mean (STD)	N(%) or Mean (STD)
All participants	394 (100)	197 (100)	197 (100)
Menopausal status and age at menopause at baseline			
Pre-menopausal	78 (19.8)	39 (19.8)	39 (19.8)
Post-menopausal	316 (80.2)	158 (80.2)	158 (80.2)
Menopausal status and age at menopause at case’s date of diagnosis			
Pre-menopausal	3 (0.8)	2 (1.0)	1 (0.5)
Post-menopausal	192 (48.7)	99 (50.3)	93 (47.2)
Age (years) at baseline	56.3 (6.2)	56.3 (6.2)	56.3 (6.2)
Age (years) at case’s date of diagnosis	68.4 (6.2)	68.4 (6.2)	68.4 (6.2)
BMI (kg/m^2^) at baseline			
< 25	279 (70.8)	136 (69.0)	136 (69.0)
≥ 25	115 (29.2)	61 (31.0)	61 (31.0)
BMI (kg/m^2^) at case’s date of diagnosis			
< 25	261 (66.2)	127 (64.5)	134 (68.0)
≥ 25	133 (33.8)	70 (35.5)	63 (32.0)
Total plasma lipid content (ng/L)	5.4 (0.8)	5.4 (0.9)	5.4 (0.8)
Smoking status at baseline			
Never	192 (48.7)	90 (45.7)	102 (51.8)
Ever	202 (51.3)	107 (54.3)	95 (48.2)
Physical activity (metabolic equivalent tasks (MET)-hour/week) at baseline			
< 35	202 (51.3)	100 (50.8)	102 (51.8)
≥ 35	192 (48.7)	97 (49.2)	95 (48.2)
Education level at baseline			
< BAC	39 (9.9)	22 (11.2)	17 (8.6)
BAC to BAC + 2	192 (48.7)	98 (49.7)	94 (47.7)
≥ BAC + 2	163 (41.4)	77 (39.1)	86 (43.7)
Personal history of benign breast disease at baseline			
No	250 (63.5)	135 (68.5)	115 (58.4)
Yes	144 (36.5)	62 (31.5)	82 (41.6)
Family history of breast cancer			
None	281 (71.3)	146 (74.1)	135 (68.5)
In first-degree relatives	61 (15.5)	31 (15.7)	30 (15.2)
In extended relatives	52 (13.2)	20 (10.2)	32 (16.3)
Parity and age at first full-term pregnancy (FFTP)			
No children	57 (14.5)	22 (11.2)	35 (17.8)
1 or 2 children and < 30 years old at FFTP	194 (49.2)	97 (49.2)	97 (49.2)
≥ 3 children and < 30 years old at FFTP	108 (27.4)	62 (31.5)	46 (23.4)
≥ 30 years old at FFTP	35 (8.9)	16 (8.1)	19 (9.6)
Total breastfeeding duration			
Never	152 (38.6)	79 (40.1)	73 (37.1)
≤ 6 months	164 (41.6)	85 (43.1)	79 (40.1)
> 6 months	78 (19.8)	33 (16.8)	45 (22.8)
Age at menarche			
< 13 years	178 (45.2)	95 (48.2)	83 (42.1)
≥ 13 years	216 (54.8)	102 (51.8)	114 (57.9)
Current use of menopausal hormone therapy at baseline			
No	234 (59.7)	119 (60.7)	115 (58.7)
Yes	158 (40.1)	77 (39.1)	81 (41.1)
Use of oral contraceptives at baseline			
Never	150 (38.1)	79 (40.1)	71 (36.0)
Ever	244 (61.9)	118 (59.9)	126 (64.0)
Adherence score to the Healthy dietary pattern			
< median	197 (50.0)	89 (45.2)	108 (54.8)
> median	197 (50.0)	108 (54.8)	89 (45.2)
Adherence score to the Western dietary pattern			
< median	197 (50.0)	98 (49.7)	99 (50.3)
> median	197 (50.0)	99 (50.3)	98 (49.7)

Quantifiable levels of PBDE-47, 99, 100, 153, and PBB-153 were detected in all samples. BDE-28 was detected in all samples except for one control with a concentration below the LOD, while BDE-154 was below the LOD in 99 samples (46 cases and 53 controls), representing 25% of the study population. The highest concentration of BFR in the overall population was observed for BDE-47 (median: 3.25 ng/L plasma; min-max: 0.39–82.65 ng/L plasma), while the lowest was observed for BDE-154 (median: 0.16 ng/L plasma; min-max: 0.03–3.76 ng/L plasma) (Table 2). Among PBDEs, we found high correlations between BDE-47, 99, 100, and 154, while BDE-28 and BDE-153 had a more modest correlation with the other congeners; in contrast, PBB-153 was very weakly correlated with plasma levels of the PBDEs considered in this study, except for PBDE-153 with which a stronger correlation was observed (rho = 0.49) (Table 3).

**Table 2:** Distribution of PBDE and PBB-153 levels (ng/L plasma and ng/gr lipids) in plasma samples collected among 394 study participants between 1994 and 1999, E3N cohort

	Cases		Controls		All		*P value
	mean (sd)	median (min-max)	mean (sd)	median (min-max)	mean (sd)	median (min-max)	
PBDE sum (ng/L plasma)	10.66 (10.46)	8.43 (2.62–100.21)	11.06 (14.22)	8.37 (3.04–165.65)	10.86 (12.47)	8.4 (2.62–165.65)	0.67
PBDE sum (ng/gr lipids)	1.96 (1.73)	1.59 (0.42–17.38)	2.07 (2.75)	1.55 (0.57–31.02)	2.01 (2.29)	1.56 (0.42–31.02)	0.74
BDE-28 (ng/L plasma)	2.14 (4.07)	0.23 (0.03–2.99)	2.30 (4.80)	0.22 (0.03–3.9)**	2.20 (4.44)	0.22 (0.03–3.9)	0.49
BDE-28 (ng/gr lipids)	0.39 (0.79)	0.04 (0.01–0.71)	0.42 (0.79)	0.04 (0.01–0.73)	0.41 (0.79)	0.04 (0.01–0.73)	0.38
BDE-47 (ng/L plasma)	1.25 (1.29)	3.26 (0.39–63.95)	1.36 (1.80)	3.21 (0.85–82.65)	1.30 (1.56)	3.26 (0.39–82.65)	0.89
BDE-47 (ng/gr lipids)	0.23 (0.21)	0.63 (0.07–11.4)	0.25 (0.35)	0.60 (0.17–15.48)	0.24 (0.29)	0.62 (0.07–15.48)	0.92
BDE-99 (ng/L plasma)	3.15 (1.49)	0.73 (0.12–14.46)	3.13 (1.55)	0.74 (0.16–47.02)	3.14 (1.52)	0.74 (0.12–47.02)	0.35
BDE-99 (ng/gr lipids)	0.58 (0.25)	0.14 (0.02–2.54)	0.59 (0.31)	0.60 (0.17–15.48)	0.58 (0.28)	0.13 (0.02–8.81)	0.27
BDE-100 (ng/L plasma)	0.19 (0.15)	0.95 (0.13–12.28)	0.22 (0.32)	0.92 (0.23–15.85)	0.21 (0.25)	0.94 (0.13–15.85)	0.92
BDE-100 (ng/gr lipids)	0.04 (0.03)	0.17 (0.03–1.84)	0.04 (0.06)	0.18 (0.04–2.97)	0.04 (0.05)	0.18 (0.03–2.97)	0.87
BDE-153 (ng/L plasma)	0.33 (0.40)	2.84 (1.06–9.93)	0.33 (0.41)	2.84 (1.35–12.47)	0.33 (0.41)	2.84 (1.06–12.47)	0.85
BDE-153 (ng/gr lipids)	0.06 (0.08)	0.53 (0.22–1.76)	0.06 (0.08)	0.53 (0.22–2.34)	0.06 (0.08)	0.53 (0.22–2.34)	0.71
BDE-154 (ng/L plasma)	4.62 (6.64)	0.16 (0.02–1.16)**	4.76 (7.52)	0.15 (0.02–3.76)**	4.69 (7.09)	0.16 (0.02–3.76)	0.61
BDE-154 (ng/gr lipids)	0.84 (1.11)	0.03 (0–0.17)	0.89 (1.46)	0.03 (0–0.7)	0.86 (1.29)	0.03 (0–0.7)	0.52
PBB-153 (ng/L plasma)	1.12 (1.59)	1.59 (0.62–57.06)	1.27 (3.62)	1.66 (0.68–66.7)	1.20 (2.80)	1.64 (0.62–66.7)	0.76
PBB-153 (ng/gr lipids)	0.21 (0.27)	0.30 (0.12–11.12)	0.24 (0.69)	0.31 (0.11–10.94)	0.22 (0.53)	0.31 (0.11–11.12)	0.62

* Kruskal–Wallis test

** minimum value = limit of detection

**Table 3:** Spearman rank correlation coefficients between PBDE and PBB-153 plasma concentrations (ng/L plasma and ng/gr lipids), E3N cohort

	PBDE28 (ng/L plasma)	PBDE28 (ng/gr lipids)	PBDE47 (ng/L plasma)	PBDE47 (ng/gr lipids)	PBDE99 (ng/L plasma)	PBDE99 (ng/gr lipids)	PBDE100 (ng/L plasma)	PBDE100 (ng/gr lipids)	PBDE153 (ng/L plasma)	PBDE153 (ng/gr lipids)	PBDE154 (ng/L plasma)	PBDE154 (ng/gr lipids)	PBB153 (ng/L plasma)	PBB153 (ng/gr lipids)
PBDE28 (ng/L plasma)	1.00													
PBDE28 (ng/gr lipids)	0.97	1.00												
PBDE47 (ng/L plasma)	0.66	0.62	1.00											
PBDE47 (ng/gr lipids)	0.63	0.66	0.96	1.00										
PBDE99 (ng/L plasma)	0.55	0.51	0.87	0.83	1.00									
PBDE99 (ng/gr lipids)	0.53	0.55	0.84	0.87	0.96	1.00								
PBDE100 (ng/L plasma)	0.63	0.59	0.92	0.87	0.81	0.76	1.00							
PBDE100 (ng/gr lipids)	0.61	0.62	0.90	0.92	0.78	0.80	0.96	1.00						
PBDE153 (ng/L plasma)	0.32	0.28	0.46	0.41	0.42	0.37	0.51	0.47	1.00					
PBDE153 (ng/gr lipids)	0.27	0.32	0.39	0.45	0.35	0.40	0.43	0.50	0.90	1.00				
PBDE154 (ng/L plasma)	0.48	0.45	0.69	0.66	0.62	0.60	0.73	0.71	0.40	0.35	1.00			
PBDE154 (ng/gr lipids)	0.45	0.47	0.65	0.68	0.59	0.62	0.69	0.72	0.36	0.39	0.97	1.00		
PBB153 (ng/L plasma)	0.05	−0.01	0.12	0.06	0.07	0.01	0.19	0.14	0.48	0.39	0.14	0.09	1.00	
PBB153 (ng/gr lipids)	0.00	0.03	0.06	0.09	0.02	0.04	0.13	0.17	0.43	0.49	0.09	0.12	0.93	1.00

Mean age at breast cancer diagnosis was 68.4 years (range 54.3–84.9 years). All cases were diagnosed after menopause except for one. Information on tumor hormone receptor expression was available in 161 cases (82%) for ER, and in 157 cases (80%) for PR. In total, 135 tumors were ER+ and 100 were PR+.

When considering concentrations of PBDEs and PBB in ng/L plasma as continuous variables, we did not find any statistically significant association between levels of PBDEs and PBB-153 and breast cancer risk. When considering these concentrations modeled in quintiles, we found no statistically significant association between the sum of PBDEs, BDE-28, 47, 99, 154, or PBB-153 and breast cancer risk in any of the models. In Models 1 and 2, women in the 3rd quintile group of BDE-100 were at lower risk of breast cancer when compared with those in the 1st quintile group (Model 2: OR 0.42, 95% CI 0.19–0.93). Similarly, when considering BDE-153, we observed a statistically significant inverse association with breast cancer risk for women in the 3rd quintile group when compared with those in the 1st quintile group in Models 1 and 2 (Model 2: OR 0.42, 95% CI 0.18–0.98) (Table 4). After stratification based on hormone receptor status, there was a statistically significant inverse association only between the 3rd quintile group of BDE-153 and ER+ breast cancer risk (OR 0.39, 95% CI 0.16–0.93) although the number of cases included in the 3rd quintile group was limited (e.g. 12 ER+ and 4 ER- cases) (**Tables S****1****and S****2**).

**Table 4:** Conditional logistic regression models to estimate the association between quintiles of PBDEs and PBB-153 plasma levels expressed in ng/L and breast cancer risk: adjusted odds ratios (ORs) and 95% confidence intervals (CIs) (E3N cohort, *N* = 394)

ng/L plasma			Model 0	P trend	Model 1	P trend	Model 2	P trend
	Number (%) controls	Number (%) cases	OR [95% CI]		OR [95% CI]		OR [95% CI]	
PBDE sum	197	197						
Q1	39(19.80)	37(18.78)	Reference	0.97	Reference	0.66	Reference	0.59
Q2	39(19.80)	36(18.27)	0.99 [0.53; 1.84]		1.09 [0.52; 2.26]		1.14 [0.54; 2.41]	
Q3	40(20.30)	38(19.29)	1.01 [0.56; 1.82]		1.00 [0.51; 1.94]		1.02 [0.52; 2.00]	
Q4	39(19.80)	44 (22.34)	1.18 [0.64; 2.18]		1.20 [0.59; 2.44]		1.20 [0.59; 2.46]	
Q5	40(20.30)	42 (21.32)	1.10 [0.58; 2.08]		1.13 [0.54; 2.39]		1.21 [0.57; 2.58]	
log-PBDE sum			0.98 [0.67; 1.42]	0.9	0.95 [0.62; 1.46]	0.83	0.97 [0.63; 1.50]	0.88
BDE-28	197	197						
Q1	39(19.80)	34 (17.26)	Reference	0.32	Reference	0.47	Reference	0.54
Q2	39(19.80)	40 (20.30)	1.16 [0.62–2.18]		1.39 [0.67–2.90]		1.36 [0.65–2.85]	
Q3	40(20.30)	34 (17.26)	0.99 [0.51–1.91]		0.92 [0.43–1.97]		0.99 [0.46–2.14]	
Q4	39(19.80)	41 (20.81)	1.18 [0.62–2.26]		1.29 [0.61–2.72]		1.32 [0.63–2.80]	
Q5	40(20.30)	48 (24.37)	1.36 [0.72–2.53]		1.33 [0.65–2.70]		1.27 [0.62–2.60]	
log-BDE-28			1.04 [0.80; 1.37]	0.75	1.00 [0.74; 1.35]	0.98	0.97 [0.72; 1.33]	0.87
BDE-47	197	197						
Q1	39(19.80)	41 (20.81)	Reference	0.72	Reference	0.81	Reference	0.72
Q2	39(19.80)	34 (17.26)	0.82 [0.43–1.56]		0.71 [0.35–1.47]		0.73 [0.35–1.53]	
Q3	40(20.30)	38 (19.29)	0.91 [0.50–1.67]		0.98 [0.49–1.95]		1.01 [0.50–2.05]	
Q4	39(19.80)	40 (20.30)	0.97 [0.53–1.79]		0.84 [0.42–1.67]		0.83 [0.41–1.69]	
Q5	40(20.30)	44 (22.34)	1.06 [0.57–1.95]		1.05 [0.52–2.11]		1.11 [0.54–2.26]	
log-BDE-47			0.94 [0.70; 1.25]	0.66	0.92 [0.66; 1.29]	0.64	0.94 [0.67; 1.32]	0.72
BDE-99	197	197						
Q1	39(19.80)	31 (15.74)	Reference	0.55	Reference	0.66	Reference	0.66
Q2	39(19.80)	45 (22.84)	1.50 [0.77–2.90]		1.30 [0.62–2.73]		1.23 [0.57–2.62]	
Q3	40(20.30)	38 (19.29)	1.27 [0.64–2.50]		1.26 [0.59–2.71]		1.32 [0.60–2.90]	
Q4	39(19.80)	41 (20.81)	1.38 [0.70–2.72]		1.20 [0.56–2.56]		1.09 [0.50–2.38]	
Q5	40(20.30)	42 (21.32)	1.36 [0.69–2.66]		1.28 [0.59–2.75]		1.29 [0.59–2.83]	
log-BDE-99			1.04 [0.78; 1.39]	0.79	1.06 [0.76; 1.48]	0.72	1.07 [0.77; 1.49]	0.69
BDE-100	197	197						
Q1	39(19.80)	49 (24.87)	Reference	0.53	Reference	0.41	Reference	0.46
Q2	39(19.80)	36 (18.27)	0.71 [0.38–1.33]		0.56 [0.27–1.17]		0.59 [0.28–1.27]	
Q3	40(20.30)	29 (14.72)	0.54 [0.28–1.05]		0.42 [0.19–0.92]		0.42 [0.19–0.93]	
Q4	39(19.80)	46 (23.35)	0.93 [0.51–1.70]		0.91 [0.46–1.80]		0.87 [0.44–1.74]	
Q5	40(20.30)	37 (18.78)	0.68 [0.35–1.30]		0.53 [0.24–1.17]		0.58 [0.26–1.28]	
Log-BDE100			0.89 [0.65; 1.20]	0.44	0.95 [0.53; 1.70]	0.85	0.97 [0.54; 1.75]	0.91
BDE-153	197	197						
Q1	39(19.80)	43 (21.83)	Reference	0.92	Reference	0.73	Reference	0.78
Q2	39(19.80)	45 (22.84)	1.08 [0.58–2.01]		1.30 [0.63–2.68]		1.28 [0.62–2.67]	
Q3	40(20.30)	22 (11.17)	0.52 [0.26–1.01]		0.40 [0.17–0.90]		0.42 [0.18–0.98]	
Q4	39(19.80)	44 (22.34)	1.02 [0.56–1.87]		1.10 [0.55–2.22]		1.12 [0.55–2.29]	
Q5	40(20.30)	43 (21.83)	0.96 [0.52–1.76]		0.85 [0.42–1.74]		0.87 [0.43–1.79]	
log-BDE-153			1.02 [0.62; 1.68]	0.94	0.95 [0.53; 1.70]	0.85	0.97 [0.54; 1.75]	0.91
BDE-154	197	197						
Q1	39(19.80)	35 (17.77)	Reference	0.47	Reference	0.86	Reference	0.86
Q2	39(19.80)	38 (19.29)	1.09 [0.56–2.11]		0.93 [0.44–1.97]		0.91 [0.43–1.96]	
Q3	40(20.30)	38 (19.29)	1.08 [0.57–2.07]		1.04 [0.50–2.15]		1.06 [0.51–2.21]	
Q4	39(19.80)	43 (21.83)	1.26 [0.66–2.43]		1.14 [0.54–2.41]		1.13 [0.53–2.40]	
Q5	40(20.30)	43 (21.83)	1.24 [0.64–2.40]		1.00 [0.47–2.13]		1.00 [0.46–2.15]	
log-BDE-154			0.99 [0.75; 1.31]	0.95	0.97 [0.70; 1.33]	0.83	0.96 [0.70; 1.33]	0.82
PBB-153	197	197						
Q1	39(19.80)	47 (23.86)	Reference	0.31	Reference	0.33	Reference	0.29
Q2	39(19.80)	41 (20.81)	0.90 [0.50–1.61]		0.73 [0.38–1.41]		0.67 [0.34–1.31]	
Q3	40(20.30)	29 (14.72)	0.58 [0.31–1.11]		0.49 [0.23–1.07]		0.48 [0.22–1.06]	
Q4	39(19.80)	51 (25.89)	1.06 [0.57–1.97]		0.96 [0.47–1.96]		0.92 [0.45–1.90]	
Q5	40(20.30)	29 (14.72)	0.58 [0.29–1.13]		0.53 [0.24–1.17]		0.50 [0.22–1.13]	
log-PBB-153			1.02 [0.62; 1.68]	0.94	0.95 [0.53; 1.70]	0.85	0.97 [0.54; 1.75]	0.91

Model 0: crude estimates;

Model 1: adjusted for total plasma lipid content (ng/L, continuous), smoking status (never vs. ever), physical activity measured in metabolic equivalent tasks (MET)-hour/week (< 35 vs. ≥35), education (≤ 12 years, 12 to 14 years, > 14 years), personal history of benign breast disease (no vs. yes), and family history of breast cancer (none, in first-degree relatives, in extended relatives), parity and age at first full-term pregnancy (FFTP) (no children, 1 or 2 children and < 30 years old at FFTP, ≥3 children and < 30 years old at FFTP, ≥ 30 years old at FFTP), total breastfeeding duration (never, ≤6 or > 6 months), age at menarche (< 13 years vs. ≥ 13 years), current use of menopausal hormone therapy (pre-menopausal, yes, no), and use of oral contraceptives (ever vs. never), menopausal status and age at menopause (pre-menopausal, menopause before age 51, menopause at age 51 or later);

Model 2: Model 1 + adherence score to the Healthy and the Western dietary patterns (above vs. below the median)

When considering concentrations of PBDEs and PBB in ng/gr of lipids in the plasma both as continuous variables and in quintiles, we did not find any statistically significant association (Table 5). We also observed no statistically significant association after stratifying based on tumor expression of estrogen receptors, while we found a positive association between the 3rd quintile group of BDE-99 and PR- breast cancer risk (OR 3.13, 95%CI 1.03–9.54) and an inverse association between women in the 2nd quintile group of PBB-153 and PR+ breast cancer risk (OR 0.34, 95% CI 0.14–0.83) (**Tables S****3****and S****4**).

**Table 5:** Conditional logistic regression models to estimate the association between quintiles of PBDE and PBB-153 plasma levels expressed as ng/gr of lipids and breast cancer risk: adjusted odds ratios (ORs) and 95% confidence intervals (CIs) (*N* = 394)

ng/gr lipids in the plasma	Number (%) controls	Number(%) cases	Model 0 OR [95% CI]	P trend	Model 1 OR [95% CI]	P trend	Model 2 OR [95% CI]	P trend
PBDE sum	197	197						
Q1	39(19.80)	44 (22.34)	Reference	0,85	Reference	0.82	Reference	0.74
Q2	39(19.80)	29 (14.72)	0.68 [0.36–1.27]		0.56 [0.27–1.14]		0.63 [0.30–1.30]	
Q3	40(20.30)	39(19.80)	0.90 [0.51–1.59]		0.92 [0.49–1.71]		0.90 [0.48–1.70]	
Q4	39(19.80)	36(18.27)	0.82 [0.45–1.50]		0.77 [0.38–1.53]		0.85 [0.42–1.72]	
Q5	40(20.30)	49 (24.87)	1.10 [0.60–2.01]		0.96 [0.48–1.91]		1.01 [0.50–2.01]	
log-PBDE sum	197	197	0.99 [0.68–1.45]	0.98	0.95 [0.62–1.46]	0.81	0.96 [0.62–1.49]	0.86
BDE-28								
Q1	39(19.80)	35 (17.77)	Reference	0.36	Reference		Reference	0.64
Q2	39(19.80)	39(19.80)	1.09 [0.58–2.02]		1.56 [0.76–3.20]	0.55	1.48 [0.71–3.09]	
Q3	40(20.30)	33 (16.75)	0.91 [0.48–1.74]		0.87 [0.41–1.86]		0.91 [0.42–1.96]	
Q4	39(19.80)	43 (21.83)	1.19 [0.65–2.17]		1.22 [0.61–2.45]		1.34 [0.67–2.71]	
Q5	40(20.30)	47 (23.86)	1.27 [0.70–2.30]		1.28 [0.65–2.50]		1.19 [0.60–2.36]	
log-BDE-28			1.05 [0.81–1.38]	0.7	0.99 [0.74–1.34]	0.9728	0.97 [0.71–1.33]	0.85
BDE-47	197	197						
Q1	39(19.80)	41 (20.81)	Reference	0.95	Reference	0.89	Reference	1,00
Q2	39(19.80)	36(18.27)	0.89 [0.49–1.61]		0.76 [0.39–1.47]		0.73 [0.37–1.44]	
Q3	40(20.30)	37(18.78)	0.89 [0.49–1.62]		0.79 [0.40–1.53]		0.74 [0.37–1.46]	
Q4	39(19.80)	43 (21.83)	1.04 [0.59–1.86]		0.97 [0.50–1.88]		0.98 [0.50–1.93]	
Q5	40(20.30)	40(20.30)	0.95 [0.51–1.79]		0.89 [0.44–1.80]		0.93 [0.45–1.92]	
log-BDE-47			0.95 [0.71–1.27]	0.71	0.92 [0.66–1.29]	0.6317	0.94 [0.66–1.32]	0.70
BDE-99	197	197						
Q1	39(19.80)	32 (16.24)	Reference	0.44	Reference	0.57	Reference	0.55
Q2	39(19.80)	38(19.29)	1.23 [0.63–2.41]		1.20 [0.58–2.49]		1.25 [0.59–2.64]	
Q3	40(20.30)	40(20.30)	1.25 [0.64–2.44]		1.27 [0.61–2.64]		1.34 [0.64–2.84]	
Q4	39(19.80)	46 (23.35)	1.47 [0.76–2.83]		1.24 [0.60–2.58]		1.20 [0.57–2.52]	
Q5	40(20.30)	41 (20.81)	1.27 [0.66–2.44]		1.27 [0.61–2.67]		1.33 [0.63–2.81]	
log-BDE-99			1.05 [0.79–1.41]	0.73	1.06 [0.76–1.47]	0.73	1.07 [0.76–1.49]	0.70
BDE-100	197	197						
Q1	39(19.80)	43 (21.83)	Reference	0.99	Reference	0.88	Reference	0.97
Q2	39(19.80)	41 (20.81)	0.97 [0.53–1.81]		0.85 [0.42–1.69]		0.99 [0.48–2.01]	
Q3	40(20.30)	29 (14.72)	0.66 [0.35–1.27]		0.58 [0.27–1.24]		0.58 [0.27–1.25]	
Q4	39(19.80)	40(20.30)	0.94 [0.52–1.72]		0.96 [0.48–1.91]		0.96 [0.48–1.93]	
Q5	40(20.30)	44 (22.34)	1.01 [0.52–1.94]		0.87 [0.41–1.84]		0.99 [0.46–2.13]	
log-BDE-100			0.90 [0.66–1.22]	0.48	0.86 [0.60–1.22]	0.40	0.87 [0.61–1.25]	0.46
BDE-153	197	197						
Q1	39(19.80)	48 (24.37)	Reference	0.85	Reference	0.84	Reference	0.87
Q2	39(19.80)	34 (17.26)	0.68 [0.36–1.30]		0.60 [0.28–1.27]		0.62 [0.29–1.31]	
Q3	40(20.30)	29 (14.72)	0.57 [0.30–1.09]		0.52 [0.24–1.12]		0.53 [0.25–1.17]	
Q4	39(19.80)	36(18.27)	0.69 [0.35–1.34]		0.75 [0.34–1.63]		0.83 [0.37–1.85]	
Q5	40(20.30)	50 (25.38)	1.00 [0.56–1.80]		0.81 [0.41–1.61]		0.80 [0.40–1.61]	
log-BDE-153			1.06 [0.64–1.76]	0.83	0.94 [0.52–1.68]	0.82	0.96 [0.53–1.73]	0.88
BDE-154	197	197						
Q1	39(19.80)	33 (16.75)	Reference	0.45	Reference	0.75	Reference	0.76
Q2	39(19.80)	38(19.29)	1.18 [0.61–2.28]		0.91 [0.43–1.92]		0.86 [0.40–1.85]	
Q3	40(20.30)	41 (20.81)	1.26 [0.63–2.51]		1.30 [0.61–2.78]		1.35 [0.63–2.90]	
Q4	39(19.80)	46 (23.35)	1.48 [0.74–2.97]		1.37 [0.63–2.98]		1.26 [0.58–2.77]	
Q5	40(20.30)	39(19.80)	1.24 [0.63–2.44]		0.99 [0.46–2.11]		0.97 [0.45–2.10]	
log-BDE-154			0.99 [0.75–1.32]	0.97	0.95 [0.69–1.31]	0.77	0.95 [0.69–1.32]	0.77
PBB-153	197	197						
Q1	39(19.80)	37(18.78)	Reference	0.59	Reference	0.80	Reference	0.74
Q2	39(19.80)	44 (22.34)	1.21 [0.63–2.32]		1.25 [0.60–2.61]		1.19 [0.56–2.56]	
Q3	40(20.30)	41 (20.81)	1.12 [0.58–2.15]		1.09 [0.52–2.30]		1.05 [0.49–2.27]	
Q4	39(19.80)	53 (26.90)	1.41 [0.73–2.72]		1.74 [0.81–3.75]		1.83 [0.83–4.04]	
Q5	40(20.30)	22 (11.17)	0.62 [0.30–1.28]		0.61 [0.27–1.38]		0.52 [0.22–1.23]	
log-PBB-153			1.06 [0.64–1.76]	0.83	0.94 [0.52–1.68]	0.83	0.96 [0.53–1.73]	0.88

Model 0: crude estimates;

Model 1: adjusted for smoking status (never vs. ever), physical activity measured in metabolic equivalent tasks (MET)-hour/week (< 35 vs. ≥35), education (≤ 12 years, 12 to 14 years, > 14 years), personal history of benign breast disease (no vs. yes), and family history of breast cancer (none, in first-degree relatives, in extended relatives), parity and age at first full-term pregnancy (FFTP) (no children, 1 or 2 children and < 30 years old at FFTP, ≥3 children and < 30 years old at FFTP, ≥ 30 years old at FFTP), total breastfeeding duration (never, ≤6 or > 6 months), age at menarche (< 13 years vs. ≥ 13 years), current use of menopausal hormone therapy (pre-menopausal, yes, no), and use of oral contraceptives (ever vs. never), menopausal status and age at menopause (pre-menopausal, menopause before age 51, menopause at age 51 or later);

Model 2: Model 1 + adherence score to the Healthy and the Western dietary patterns (above vs. below the median)

In a sensitivity analysis, we repeated the analyses after excluding the case diagnosed before menopause and her matched control, and the results remained unchanged (data not shown). In addition, compared to the main analyses, the results remained virtually unchanged in terms of magnitude and statistical significance in sensitivity analyses adjusting for the evolution of women’s body size or BMI variation over the 5 years preceding blood sampling (data not shown).

In analyses stratified according to BMI (< 25 and  ≥25 kg/m^2^) and considering PBDE and PBB concentrations in ng/L plasma, women in the 3rd quintile group of BDE-100 with a BMI < 25 kg/m^2^ were at lower risk of breast cancer (OR 0.33, 95% CI 0.12–0.90, compared with the 1st quintile group). Among women with a BMI ≥25 kg/m^2^, we found an inverse association with breast cancer risk among women in the 3rd quintile group of BDE-153 (OR 0.05, 95% CI < 0.00–0.62) and among those in the 3rd quintile group of PBB-153 (OR 0.13, 95% CI < 0.02–0.87). On the other hand, when considering concentrations of PBDEs and PBB in ng/gr of lipids in the plasma, we observed a statistically significant inverse relationship only for the 2nd and 3rd quintile groups of BDE-153 and only in women with a BMI ≥25 kg/m^2^ (OR 0.09, 95% CI 0.01–0.08 and OR 0.07, 95% CI 0.01–0.58, respectively) (**Tables S****5****and S****6**).

## Discussion

In this nested case-control study of non-professionally exposed French women, we found no evidence of an association between plasma levels of PBDEs and PBB-153 and breast cancer risk, although when running the analyses with concentrations expressed as ng/L plasma, there was an inverse association between the 3rd quintile group of BDE-100 and BDE-153 and the risk of breast cancer.

To date, few studies have investigated the association between internal levels of PBDEs and PBB and breast cancer risk, and they led to inconsistent results. One study including 902 women with invasive breast cancer and 936 controls from the California Teachers Study investigated the association between serum levels of BDE-47, − 100, and − 153 and the risk of breast cancer, suggesting no association [14].

A hospital-based study conducted among native Alaskan women, including invasive and in situ breast cancer as cases (*n* = 75) and women with diagnoses of benign breast conditions as controls (*n* = 95), failed to find any statistically significant association between serum levels of BDE-47 and breast cancer risk [15]. Two studies have investigated the associations between levels of PBDEs in breast adipose tissue and the risk of breast cancer. The first one, a Californian hospital-based study, which enrolled women with histologically-confirmed invasive breast cancer as cases (*n* = 78) and women with benign histological changes as controls (*n* = 56), provided no evidence of an association between adipose tissue concentrations of BDE-47, − 99, − 100, − 153, and − 154 and breast cancer risk [16]. The second was a hospital-based study conducted in China (Shantou, Chaoshan area) and included 209 cases and 165 controls. In multivariable models adjusted for age and menarcheal age, adipose tissue levels of BDE-47, − 71, − 99, − 100, − 183 and − 209 were independently and positively associated with breast-cancer risk [17]. Some major differences need to be considered when comparing these previous findings with our results. First, these studies had a retrospective case-control design, implying that the exposure level was measured at the time of diagnosis (or after diagnosis), while in the present study blood samples were drawn before breast cancer diagnosis. Moreover, in the latter two studies, PBDE levels were measured in adipose tissue, while in our study only levels measured in blood were available.

Of note, a nested case-control study was conducted based on 51 cases of breast cancer and 202 controls selected among women residents in Michigan during the industrial accident in 1973, which led to a high contamination of the local food chain with PBB [18]. This study found an OR of 2.60 (95% CI 0.93–7.27) for breast cancer incidence in women with a serum level of PBB ≥10 ng/mL compared with women with PBB ≤1 ng/mL. Although not statistically significant, these results confirmed those found in two previous studies in the same population that were performed within a shorter time interval after the accident. Henderson et al. (follow-up through 1992) and Hoque et al. (follow-up through 1993) found non-statistically significant positive associations between PBB exposure and breast cancer risk [19, 20]. These studies differed from ours in the fact that the Michigan population represents a highly exposed group, with circulating levels 1000-fold higher compared with those found in our study population.

The exposure levels of PBDEs in our study were substantially lower compared to other previous studies with a similar blood collection time. These diverging biomonitoring profiles are aligned with the higher production of BFRs in US (e.g. 3–7 folds) in comparison to Europe between late 70s and 90s [6]. For instance, the median concentration of BDE-153 was 6.5–7.2 ng/g in adipose tissue from US [16] and 0.53 ng BDE-153 /g lipid of plasma from France in the 90s (present study). These lower levels of exposure in France are further supported by subsequent studies conducted in US, finding a median of 4.8 ng/g lipid of serum between 2003 and 2004 [32], or in China, showing a range between 26.02–31.02 ng/g in adipose tissue between 2014 and 2016 [17], whereas in France a median of 0.75 ng/ g lipid of serum has been reported in the last biomonitoring study (2014–2016) [33]. Moreover, our improved analytical method substantially increased the sensitivity and the number of detected samples, for instance, our mean LOD for BDE153 was 0.1 ng/L whereas previous studies have reported LOD of 17 ng/L [16].

Overall, we interpret the results from our study as null findings. However, it should be noted that when running the analyses with the exposure expressed as ng/L of plasma, we observed a non-linear inverse association between levels of PBDE-100 and -153 and breast cancer risk; nevertheless, this association was not statistically significant when considering concentrations in ng/gr of lipids in the plasma. With few exceptions, stratification by tumor hormone receptor expression and BMI categories yielded similar results as the main analyses. Therefore, given the relatively small number of cases upon which these estimates are based, the inconsistency in the results obtained for other PBDEs included in the study, and the numerous statistical analyses that were conducted, it is probable that these findings are due to chance.

When interpreting these results, a limitation of our study that should be taken into consideration is that only a single measurement of PBDE and PBB-153 concentrations was available; we thus had no information on the true trajectories of exposure or on exposure levels during sensitive periods of breast development. The present study was conducted with a population exposed to low background levels of PBDEs and PBBs limiting the conclusion to the low end of the population dose-response curve. Moreover, previous studies have suggested that certain genetic factors may modify the associations between exposure to EDCs and risk of breast cancer [34], but no genetic information was available in our study to investigate this hypothesis. Additionally, PBDE and PBB-153 levels were measured in blood plasma and no information on breast adipose tissue levels of PBDEs and PBB-153 were available. Despite the fact that blood biomarkers of lipophilic pollutants are acknowledged to be good predictors of levels in adipose tissue, many factors may affect the partition between lipids from both compartments. For instance, the average partition adipose tissue:serum of PBDEs in lipid basis was estimated around 0.5 for the congeners BDE-28, − 47 and − 99 and between 0.8 and 1.1 for BDE-153, − 154 and − 183 in Chinese women [35]. This imperfect partition may hamper the interpretation of risk models when using circulating biomarkers as proxies of adipose tissue estimates, resulting, in some cases, in associations in opposite directions [36]. Nonetheless, the systematic comparison between models with lipid-adjusted and wet-basis biomarkers did not yield substantial modifications of risk estimates, thus ruling out more complex underlying associations. We used the parent forms of PBDEs as biomarkers of internal exposure, while little is known about the potential adverse health effects of their hydroxylated forms as products of biotransformation metabolism, which have shown exert estrogenic effects [37]. Thus, one hypothesis to be deeper investigated for explaining the observed reverse associations would be the potential protective effect of adipose tissue by sequestrating the parent compounds and more biologically active hydroxylated metabolites, preventing the release into circulation and thus the exposure of carcinogenic cells [38]. Finally, we would also emphasize that, despite the fact that the biomarkers were mostly detected in all samples, we were not able to detect BDE-154 in 25% of samples; thus, the related results should be considered with caution due to the risk of bias associated to the substitution of non-detected samples by ½ LOD.

Nevertheless, our study presents several strengths. Thanks to the long follow-up duration of the E3N cohort, we were able to prospectively investigate the long-term health effects of plasma concentrations of PBDEs and PBB-153. Extensive information on the main breast cancer risk factors, such as reproductive history, use of hormonal treatments, anthropometric characteristics, and lifestyle-related risk factors, was collected prospectively in the cohort and thus at the same time for cases and controls. Finally, the availability of information on breast cancer expression of estrogen and progesterone receptors allowed us to better characterize the tumors and to perform stratified analyses.

## Conclusion

Our results suggest no clear association between internal levels of PBDEs and PBB-153 and risk of breast cancer in postmenopausal women. Although our results are similar to those found in previous research, they need to be carefully interpreted, taking into account study limitations due to the limited number of women included in the study, the lack of information concerning genetic susceptibility, and the unavailability of exposure assessment during critical windows of susceptibility for breast cancer. More studies are warranted to further investigate the relationships between PBDE and PBB exposure and breast cancer risk, in particular through deeper investigation related to the link between those markers and lipids, and documenting exposure levels for potentially active PBDE metabolites.

## Supplementary information

**Additional file 1 Table S1.** Multinomial regression models to estimate the association between quintiles of PBDEs and PBB-153 plasma level expressed as ng/L and breast cancer risk stratified according to hormone estrogen receptor status: adjusted odds ratios (ORs) and 95% confidence intervals (CIs). **Table S2.** Multinomial regression models to estimate the association between quintiles of PBDEs and PBB-153 plasma level expressed as ng/L and breast cancer risk stratified according to hormone progesteron receptor status: adjusted odds ratios (ORs) and 95% confidence intervals (CIs). **Table S3.** Multinomial regression models to estimate the association between quintiles of PBDEs and PBB-153 plasma level expressed as ng/gr of lipids and breast cancer risk stratified according to hormone estrogen receptor status: adjusted odds ratios (ORs) and 95% confidence intervals (CIs). **Table S4.** Multinomial regression models to estimate the association between quintiles of PBDEs and PBB-153 plasma level expressed as ng/gr of lipids and breast cancer risk stratified according to hormone progesteron receptor status: adjusted odds ratios (ORs) and 95% confidence intervals (CIs). **Table S5.** Conditional logistic regression models to estimate the association between quintiles of PBDEs and PBB-153 plasma levels expressed as ng/L and breast cancer risk stratified according to the body mass index (BMI): adjusted odds ratios (ORs) and 95% confidence intervals (CIs) **Table S6.** Conditional logistic regression models to estimate the association between quintiles of PBDEs and PBB-153 plasma levels expressed as ng/gr of lipids and breast cancer risk stratified according to the body mass index (BMI): adjusted odds ratios (ORs) and 95% confidence intervals (CIs)
